# An Empirical Analysis of Rough Set Categorical Clustering Techniques

**DOI:** 10.1371/journal.pone.0164803

**Published:** 2017-01-09

**Authors:** Jamal Uddin, Rozaida Ghazali, Mustafa Mat Deris

**Affiliations:** Faculty of Computer Science and Information Technology, Universiti Tun Hussein Onn, Johor, Malaysia; Jiangnan University, CHINA

## Abstract

Clustering a set of objects into homogeneous groups is a fundamental operation in data mining. Recently, many attentions have been put on categorical data clustering, where data objects are made up of non-numerical attributes. For categorical data clustering the rough set based approaches such as Maximum Dependency Attribute (MDA) and Maximum Significance Attribute (MSA) has outperformed their predecessor approaches like Bi-Clustering (BC), Total Roughness (TR) and Min-Min Roughness(MMR). This paper presents the limitations and issues of MDA and MSA techniques on special type of data sets where both techniques fails to select or faces difficulty in selecting their best clustering attribute. Therefore, this analysis motivates the need to come up with better and more generalize rough set theory approach that can cope the issues with MDA and MSA. Hence, an alternative technique named Maximum Indiscernible Attribute (MIA) for clustering categorical data using rough set indiscernible relations is proposed. The novelty of the proposed approach is that, unlike other rough set theory techniques, it uses the domain knowledge of the data set. It is based on the concept of indiscernibility relation combined with a number of clusters. To show the significance of proposed approach, the effect of number of clusters on rough accuracy, purity and entropy are described in the form of propositions. Moreover, ten different data sets from previously utilized research cases and UCI repository are used for experiments. The results produced in tabular and graphical forms shows that the proposed MIA technique provides better performance in selecting the clustering attribute in terms of purity, entropy, iterations, time, accuracy and rough accuracy.

## 1 Introduction

The grouping of objects having similar characteristics in the same cluster and having dissimilarity into different clusters is the keen objective of clustering. Moreover, clustering can segment large heterogeneous data sets into smaller homogeneous subsets which is easily managed, separately modeled and analyzed [1]. Clustering has been utilized for various data mining tasks like data summation and classification. In many areas such as research and development [2], marketing [3], medicine [4], nuclear science [5], software engineering [6] and radar scanning [7] clustering techniques are used. Large scale research and development planning is identified by Mathieu and Gibson [2] using cluster analysis as a part of a decision support tool to participate and determine resource allocation. Wu et al. [4] developed a specific clustering algorithm designed for handling the gene data complexity. Wong etal. [5] presented an approach for positron emission tomography (PET) that is used to segment tissues in a nuclear medical imaging. Radar signals are segmented in marine objects and scanning land by Haimov et al. [7] using cluster analysis.

All these mentioned algorithms only deal those databases having attributes with numeric domains. Unlike numerical data, categorical data have multi-valued attributes in which the horizontal co- occurrences (common value for the objects) as well as the vertical co-occurrences (common value for the attributes) are required to be examined [4]. Thus, a similarity for the attributes can be defined for common objects, common values and the association between two. To handle categorical data clustering issue, Huang [1], Gibson et al. [8], Guha et al. [4] and Dempster et al. [9] contributed up to some extent but their techniques cannot deal with uncertainty [10]. Uncertainty is when there is no sharp boundary between clusters and it has become an integral part of most of the real world applications nowadays.

The rough set theory, proposed by Pawlak in 1982 [11] can be seen as a reliable mathematical approach towards the uncertainty. The first attempt on rough set based technique to select clustering attribute is proposed by Mazlack et al. [12]. They proposed two techniques, i.e., Bi-Clustering(BC) and Total Roughness(TR) techniques. Parmar et al. [13] proposed an algorithm Minimum-Minimum Roughness (MMR) in 2007 as one of the most successful pioneering rough clustering techniques. The generalizabilty and clusters purity of these techniques are still an issue as they can be applied only for a very special data set and objects in different class appear in one clusters, respectively [14]. Hence in 2010, Herawan et al. [15] proposed a technique to selecting clustering attribute called maximum dependency of attributes (MDA) which take into account the dependency of attributes in an information system using rough set theory. In 2013, Hassanein and Elmelegy [16] proposed a better and new approach for selecting clustering attribute called maximum significance attribute (MSA). This technique is based on the significance of attributes using rough set theory in an information system. Both MDA and MSA outperformed their predecessors approaches like BC, TR and MMR in terms of purity, computational complexity and rough accuracy up to certain level. However, MDA and MSA techniques have some limitations and issues while dealing with some special data sets in selecting the best clustering attribute. Moreover, these techniques have certain pros and cons which are explored in this study.

It is well known that the MSA and MDA approaches works to find their best possible clustering attribute on basis of maximum dependency and significance degrees respectively. Accordingly the following questions may arise when employing MSA and MDA techniques to any data set.

1. What if attributes have zero dependency and significance degree?
2. What if attributes have same degree of dependencies or significance?
3. What if the techniques select different attributes as their best clustering attribute?

First two questions illustrate the limitations of MDA and MSA techniques where they found difficulty in selecting or failed to select the best clustering attribute. While last question deal with exploring some useful pros and cons of both approaches. In the light of above research questions and limitations of existing techniques, a new rough clustering approach called Maximum Indiscernible Attribute (MIA) is proposed which uses the rough set indiscernibility relations for finding best clustering attribute. A set of objects can be characterized using rough set approach in terms of attribute values [17] and the partitions induced by indiscernibility relation of an attribute shows clusters obtained. Therefore, the number of clusters can be computed by finding cardinality of indiscernibility relation of any attribute. The number of clusters have also been used for evaluating clusters internally in [18] and [19]. Moreover in this paper, the effect of number of clusters on purity and entropy is also explored using propositions to validate the proposed approach.

The MIA technique selects the best clustering attribute having maximum cardinality of attribute’s indiscernibility relation. Therefore, it takes into account only the domain knowledge of any data set, hence it has lesser computational complexity as compare to MDA and MSA techniques. Similarly, experimental results reveal that the MIA technique outperformed MDA and MSA techniques for all evaluation measures like purity, entropy, accuracy and rough accuracy.

The rest of this paper is organized as follows. Section 2 describes the rough set theory. Section 3 illustrates the proposed MIA technique and related propositions. The analysis of MDA and MSA techniques with some useful propositions and evaluation measures are presented in Section 4. Section 5 presents the experimentation, comparison of the techniques in light of each research questions. Section 6 discusses the experimental results. Finally, Section 7 concludes the study.

## 2 Pawlak’s Rough Set

In early 1980s Zdzislaw Pawlak introduced Rough set theory as a new mathematical tool to deal with vagueness and uncertainty [20]. In the presence of uncertainty, rough set theory aids decision making [15]. Rough set theory does not need any preliminary or additional information about data, such as probability distribution in statistics, basic probability assignment in the Dempster-Shafer theory, or grade of membership or the value of possibility in fuzzy set theory [20]. With every object of the universe of discourse some information (data, knowledge) is associated, this founded assumption of rough set theory. This can be understood by letting a group of patients suffering from a specific disease. Information like name, age, address, temperature and blood pressure is contained in each patient’s associated data file. Elementary granules of knowledge about patients (or types of patients) can be understood as same symptoms patients are indiscernible (similar) in view of the available information and can be classified in blocks. These blocks are called elementary sets or concepts, and can be considered as initial blocks of knowledge about patients.

This bring motivation for rough set theory that it represents subsets of a universe in terms of equivalence classes of a clustering of the universe. The concept of rough set theory is used here in term of data containing in an information system. For the representation of objects in terms of their attribute values the information system notation provides a convenient tool. Rough set information system is a 4-tuple (quadruple) *S* = (*U*, *A*, *V*, *δ*), where *U* is a non-empty finite set of objects, *A* is a non-empty finite set of attributes, *V* = ⋃_*a* ∈ *A*_ *V*_*a*_, *V*_*a*_ is the domain(value set) of attribute *a*, *δ*: *U* × *A* → *V* is a function such that *δ*(*u*, *a*) ∈ *V*_*a*_ for every (*u*, *a*) ∈ *U* × *A*, called information function [11].

With every set *X* ⊆ *U* two crisp sets can be associated, called the lower and the upper approximation of *X*. The notions of lower and upper approximations of a set can be defined as follows [11].

For *T* ⊆ *A*, the T-lower approximation of *X*, denoted by $\underline{T}\left(X\right)$ and T-upper approximation, denoted by $\bar{T}\left(X\right)$ of X, respectively. The lower approximation of X is the union of all elementary set which are included in X.

$$\begin{array}{c} \underline{T}\left(X\right)=\left\{x\in U|{\left[x\right]}_{T}\subseteq X\right\} \end{array}$$ (1)

Whereas the upper approximation of *X* is the union of all elementary set which have non-empty intersection with X, that is,

$$\begin{array}{c} \bar{T}\left(X\right)=\left\{x\in U|{\left[x\right]}_{T}\cap X\neq \phi \right\} \end{array}$$ (2)

In other words the lower approximation of a set is the set of all elements that surely belongs to X, whereas the upper approximation of X is the set of all elements that possibly belong to X. The difference of the upper and the lower approximation of X is its boundary region. Obviously a set is rough if it has non empty boundary region; otherwise the set is crisp. The T-boundary region of X will be referred as set,

$$\begin{array}{c} BN_{T}\left(X\right)=\bar{T}\left(X\right)-\underline{T}\left(X\right). \end{array}$$ (3)

The accuracy of approximation (rough accuracy) of any subset *X* ⊆ *U* with respect to *T* ⊆ *A*, denoted *η*_*T*_(*X*) is measured by,

$$\begin{array}{c} \eta_{T}\left(X\right)=\frac{|\underline{T}\left(X\right)|}{|\bar{T}\left(X\right)|} \end{array}$$ (4)

Where |*X*| denotes the cardinality of *X*. For empty set *ϕ*, we define *η*_*T*_(*ϕ*) = 1. Obviously 0 ≤ *η*_*T*_(*X*) ≤ 1. If *X* is a union of some equivalence classes of **U**, then *η*_*T*_(*X*) = 1. Thus, the set *X* is crisp with respect to *T*. And, if *X* is not a union of some equivalence classes of *U*, then *η*_*T*_(*X*) < 1. Thus, the set *X* is rough(imprecise) with respect to *S* [11]. This means that higher the accuracy of approximation of any subset *X* ⊆ *U* is the more precise (the less imprecise) of it self [15].

## 3 Maximum Indiscernible Attribute (MIA)

In this section, we will present the proposed technique, which we refer to as maximum indiscernible attribute (MIA). Rough indiscernibility relation of attribute(s) which is the domain knowledge of information systems is taken into account for MIA technique. Let *T* be any subset of *A*, two elements *x*, *y* ∈ *U* is said to be T-indiscernible (indiscernible by the set of attribute *T* ⊆ *A* in *S*) if and only if *δ*(*x*, *t*) = *δ*(*y*, *t*) for every *t* ∈ *T*. Obviously, every subset *T* of *A* induces unique equivalence indiscernibility relation and unique clustering denoted by *IND*(*T*). The clustering of *U* induced by *IND*(*T*) in *S* denoted by *U*/*T* and the equivalence class in the clustering *U*/*T* containing *x* ∈ *U*, denoted by [*x*]_*T*_. The cardinality of indiscernibility relation of an attribute(s) will show the number of clusters obtained by that attribute and can be evaluated as,

$$\begin{array}{c} card(IND(T))=|IND(T)| \end{array}$$ (5)

**Fig 1:**
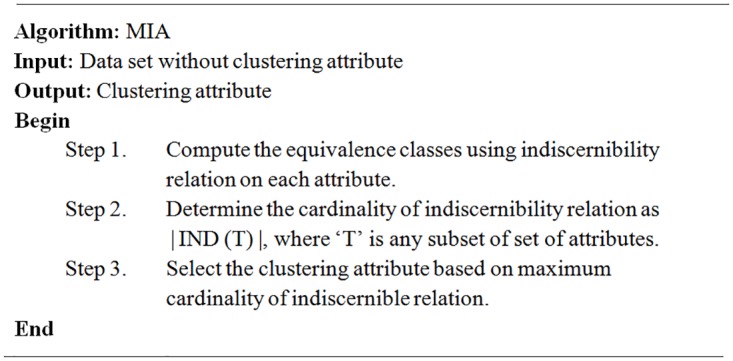
The MIA Algorithm.

The pseudo-code of the MIA algorithm is illustrated in Fig 1. This algorithm comprises of three main steps. The first step deals with the computation of indiscernibility relations for each attribute. The second step deals with the determination of each attribute’s indiscernibility relation cardinality. This cardinality can be determined using Eq (5). In the last step, when each cardinality is computed, then the clustering attribute will be selected based on maximum cardinality. If the highest value of cardinality of indiscernibility relation is same with other, then it is recommended to take into account the pair of attributes that are tied and so on, until the tie is broken. An equivalence relation of selected attribute(s) will give the clusters obtained.

The cardinality of indiscernibility relation shows the number of clusters created. This idea of selecting attribute having maximum cardinality of indiscernibility relation is based on a study which claims that high purity is easy to achieve when the number of clusters is large [21]. Similarly, the number of clusters obtained at each step of the clustering process is an indicator of the success fullness of a clustering approach [22]. Though, large number of clusters means more cohesive and low coupled clusters are created [23], this provide justification that the higher the cardinality of indiscernibility relation of attribute(s), the more accurate for selecting clustering attribute.

We first present the relation between the properties of roughness of a subset *X* ⊆ *U* with the cardinality of indiscernibility relation of two attributes as stated in Proposition 1. Generalization of Proposition 1 is given in Proposition 2. Moreover, Proposition 3 and Proposition 4 illustrates the effect of the number of clusters on purity and entropy of clustering respectively.

**Proposition 1** *Let* *S* = (*U*, *A*, *V*, *δ*), *be an information system, let* *L* *and* *M* *be any subset of* *A*. *If* *L* *has more indiscernible elements than* *M*, *that is* |*IND*(*M*)| ≤ |*IND*(*L*)| *then*,

$$\begin{array}{c} \alpha_{M}\left(X\right)\leq \alpha_{L}\left(X\right), \end{array}$$

*for every* *X* ⊆ *U*.

**Proof:** Let *L* and *M* be any subsets of *A* in information system *S* = (*U*, *A*, *V*, *δ*). From the hypothesis, we have *IND*(*L*) ⊆ *IND*(*M*). Furthermore, the clustering *U*/*L* is finer than that *U*/*M*, thus, it is clear that any equivalence class induced by *IND*(*M*) is a union of some equivalence class induced by *IND*(*L*). Therefore, for every *x* ∈ *X* ⊆ *U*, we have

[*x*]_*L*_ ⊆ [*x*]_*M*_.

And hence, for every *X* ⊆ *U*, we have

$\underline{M}\left(X\right)\subseteq \underline{L}\left(X\right)\subset X\subset \bar{L}\left(X\right)\subseteq \bar{M}\left(X\right).$

Consequently,

$\alpha_{M}\left(X\right)=\frac{|\underline{M}\left(X\right)|}{|\bar{M}\left(X\right)|}\leq \frac{|\underline{L}\left(X\right)|}{|\bar{L}\left(X\right)|}=\alpha_{L}\left(X\right).$

The generalization of Proposition 1 is given below.

**Proposition 2** *Let* *S* = (*U*, *A*, *V*, *δ*) *be an information system, and let* *L*_1_, *L*_2_, …, *L*_*n*_ *and* *M* *be any subsets of* *A*. *If* |*IND*(*M*)| ≤ |*IND*(*L*_*j*_)|, *for* *j* = 1, 2, …, *n*, *then*

*α*_*M*_(*X*) ≤ *α*_*L*_*n*__(*X*) ≤ *α*_*L*_*n*−1__(*X*)… ≤ *α*_*L*_2__(*X*) ≤ *α*_*L*_1__(*X*),

*for every* *X* ⊆ *U*.

**Proof:** Let *L*_1_, *L*_2_, …, *L*_*n*_ and *M* be any subsets of *A* in an information system *S* = (*U*, *A*, *V*, *δ*). From the hypothesis and follows from Proposition 1, we have

*α*_*M*_(*X*) ≤ *α*_*L*_1__(*X*)

*α*_*M*_(*X*) ≤ *α*_*L*_2__(*X*)

.

.

.

*α*_*M*_(*X*) ≤ *α*_*L*_*n*__(*X*).

Since, |*IND*(*M*)| ≤ |*IND*(*L*_*n*_)| ≤ |*IND*(*L*_*n*−1_)|… ≤ |*IND*(*L*_2_)| ≤ |*IND*(*L*_1_)|, then

[*x*]_*L*_*n*__ ⊆ [*x*]_*L*_*n*−1__

[*x*]_*L*_*n*−1__ ⊆ [*x*]_*L*_*n*−2__

.

.

.

[*x*]_*L*_2__ ⊆ [*x*]_*L*_1__.

Obviously,

*α*_*M*_(*X*) ≤ *α*_*L*_*n*__(*X*) ≤ *α*_*L*_*n*−1__(*X*) ≤ … ≤ *α*_*L*_2__(*X*) ≤ *α*_*L*_1__(*X*).

**Proposition 3** *Increasing number of clusters maximizes purity*.

**Proof:** The extent to which a cluster contains objects of a single class is called purity [24]. After calculating the class distribution of the data for each cluster, i.e., for cluster *x* we compute *P*_*xy*_, the probability that a member of cluster *x* belongs to class *y* as *P*_*xy*_ = *c*_*xy*_/*c*_*x*_, where *c*_*x*_ is the number of objects in cluster *x* and *c*_*xy*_ is the number of objects of class *y* in cluster *x*. The purity of cluster *x* is *P*_*x*_ = *max*_*y*_*P*_*xy*_ and the overall purity of a clustering is,

$$\begin{array}{c} Purity=\sum_{x=1}^{k}\frac{c_{x}}{c}P_{x} \end{array}$$ (6)

Eq (6) of purity can be simplified to,

$$\begin{array}{c} purity=\sum_{i=1}^{k}\frac{c_{x}}{c}P_{x}=\sum_{i=1}^{k}\frac{c_{xy}}{c} \end{array}$$ (7)

If we consider the worst possible case i.e. in selected best attributes each cluster in them has just single object correctly classified to particular class, than Eq (7) gives,

$$\begin{array}{c} purity\left(\left(k\right)\text{clusters}\right)=\frac{1}{c}+\frac{1}{c}+...+\frac{1}{c}=\frac{k}{c} \end{array}$$

$$\begin{array}{c} purity\left(\left(k-1\right)\text{clusters}\right)=\frac{1}{c}+\frac{1}{c}+...+\frac{1}{c}=\frac{k-1}{c} \end{array}$$

$$\begin{array}{c} purity\left(\left(k-2\right)\text{clusters}\right)=\frac{1}{c}+\frac{1}{c}+...+\frac{1}{c}=\frac{k-2}{c} \end{array}$$

Which shows that reducing the number of clusters minimize the purity of clusters because, $\frac{k}{c}>\frac{k-1}{c}>\frac{k-2}{c}$ is always true. And if we consider best possible case i.e. all objects of clusters of selected attribute correctly classified to particular class, than Eq (7) shows,

$$\begin{array}{c} purity\left(\left(k\right)\text{clusters}\right)=\frac{a}{c}+\frac{b}{c}+...+\frac{x}{c}=\frac{a+b+..+x}{c}=1 \end{array}$$

$$\begin{array}{c} purity\left(\left(k-1\right)\text{clusters}\right)=\frac{d}{c}+\frac{e}{c}+...+\frac{y}{c}=\frac{d+e+..+y}{c}=1 \end{array}$$

$$\begin{array}{c} purity\left(\left(k-2\right)\text{clusters}\right)=\frac{f}{c}+\frac{g}{c}+...+\frac{z}{c}=\frac{e+f+..+z}{c}=1 \end{array}$$

In this case we always get purity 1 which means that for ideal case, reducing the number of clusters has no effect on the purity of clusters. As long as all objects of clusters of selected attribute are correctly classified to particular class it will always give 100percent purity.

**Proposition 4** *Increasing number of clusters minimizes entropy*.

**Proof:** The degree to which each cluster consists of objects of a single class is called Entropy [24]. Smaller the entropy is, better will be the clustering performance. Using the class distribution and previous terminology, the entropy of each cluster *x* is calculated using the standard formula,

$$\begin{array}{c} E_{x}=-\sum_{y=1}^{L}P_{xy}log_{2}P_{xy} \end{array}$$ (8)

Where *L* is the number of classes. The total entropy for a set of clusters is calculated as the sum of entropies of each cluster weighted by the size of each clusters, that is,

$$\begin{array}{c} E=\sum_{x=1}^{k}\frac{c_{x}}{c}E_{x} \end{array}$$ (9)

Where *k* is the number of clusters and *m* is the total number of data points.

Eq (8) of entropy can be simplified to,

$$\begin{array}{c} E=\sum_{x=1}^{k}\frac{c_{x}}{c}E_{x}=-kL\frac{c_{xy}}{c}log_{2}\frac{c_{xy}}{c_{x}} \end{array}$$ (10)

If in selected group of clusters, each cluster in them has just single object correctly classified to particular class then *c*_*xy*_ = *c*_*sy*_ = *c*_*ty*_ = 1. For this worst possible case for any clustering solution, the Eq (10) results,

$$\begin{array}{c} Entropy\left(\left(k\right)\text{clusters}\right)=-kL\frac{1}{c}log_{2}\frac{1}{c_{x}}=-\frac{kL}{c}log_{2}\frac{1}{c_{x}} \end{array}$$

$$\begin{array}{c} Entropy\left(\left(k-1\right)\text{clusters}\right)=-\left(k-1\right)L\frac{1}{c}log_{2}\frac{1}{c_{s}}=-\frac{(k-1)L}{c}log_{2}\frac{1}{c_{s}} \end{array}$$

$$\begin{array}{c} Entropy\left(\left(k-2\right)\text{clusters}\right)=-\left(k-2\right)L\frac{1}{c}log_{2}\frac{1}{c_{t}}=-\frac{(k-2)L}{c}log_{2}\frac{1}{c_{t}} \end{array}$$

For inequality *k* > *k* − 1 > *k* − 2 we have *e*_*x*_ < *e*_*y*_ < *e*_*z*_, because by reducing number of clusters will increase the size of each cluster. Hence, $-\frac{kL}{c}log_{2}\frac{1}{c_{x}}<-\frac{(k-1)L}{c}log_{2}\frac{1}{c_{s}}<-\frac{(k-2)L}{c}log_{2}\frac{1}{c_{t}}$ is always true. Hence, it shows that the entropy will minimize for increasing number of clusters.

Ideally, if each cluster will contain elements from only one class then entropy is 0 [24]. Considering this best possible case for any clustering solution, where all objects inside clusters are correctly classified to a particular expert cluster then *c*_*xy*_ = *c*_*x*_, *c*_*sy*_ = *c*_*s*_, *c*_*ty*_ = *c*_*t*_. Hence, Eq (10) simplified to,

$$\begin{array}{c} Entropy\left(\left(k\right)\text{clusters}\right)=-kL\frac{1}{c}log_{2}\frac{c_{xy}}{c_{x}}=-\frac{kLc_{xy}}{c}log_{2}1=0 \end{array}$$

$$\begin{array}{c} Entropy\left(\left(k-1\right)\text{clusters}\right)=-\left(k-1\right)L\frac{1}{c}log_{2}\frac{c_{sy}}{c_{s}}=-\frac{\left(k-1\right)Lc_{sy}}{c}log_{2}1=0 \end{array}$$

$$\begin{array}{c} Entropy\left(\left(k-2\right)\text{clusters}\right)=-\left(k-2\right)L\frac{1}{c}log_{2}\frac{c_{ty}}{c_{t}}=-\frac{\left(k-2\right)Lc_{ty}}{c}log_{2}1=0 \end{array}$$

In this case we always get entropy 0 because reducing the number of clusters has no effect on the entropy of clusters as long as all objects of clusters are correctly classified to only one expert cluster.

## 4 Performance Comparison

The performance of two existing rough set techniques in clustering categorical data that is MDA and MSA is investigated. In the subsequent subsections we briefly illustrates the analysis of these techniques, related propositions and evaluation metrics that are employed in this study.

### 4.1 Maximum dependency attributes (MDA)

Based on rough set theory using the dependency of attributes in information systems, Herawan et al. [15] in 2010 proposed MDA technique for selecting the clustering attribute. Let *S* = (*U*, *A*, *V*, *δ*) be an information system and let *P* and *Q* be any subsets of *A*. Degree of dependency of attribute *Q* on attributes *P*, denoted *P* ⇒_*k*_ *Q*, is defined by,

$$\begin{array}{c} k=\frac{\Sigma_{x\in U/Q}\left|\underline{P}\left(X\right)\right|}{\left|U\right|} \end{array}$$ (11)

Obviously, 0 ≤ *k* ≤ 1. Attribute *Q* is said to be depends totally (in a degree of *k*) on the attribute *P* if *k = 1*. Otherwise, *Q* depends partially on *P*. The maximum degree of dependency of attributes is the more accurate (higher of accuracy of approximation) for selecting clustering attribute [25]. If the highest value of an attribute is the same with other attributes, then it is recommended to look at the next highest MDA inside the attributes that are tied and so on until the tie is broken.

### 4.2 Maximum significance of attributes (MSA)

In 2013, Hassanein and Elmelegy [16] proposed MSA technique for selecting clustering attribute. It uses the rough set theory concept of significance of attributes in information systems. Suppose significance of single attribute *a*_*i*_ ∈ A related to *a*_*j*_ ∈ A,

$$\begin{array}{c} \sigma_{a_{j}}\left(a_{i}\right)=\gamma_{A^{{}^{\prime }}}\left(a_{j}\right)-\gamma_{A^{{}^{\prime \prime }}}\left(a_{j}\right), \end{array}$$ (12)

where *A*′ = *A* − {*a*_*j*_}, *A*′′ = *A*′ − {*a*_*i*_}. Here, the attribute having maximum degree of significance is selected as the best clustering attribute. If the highest value of an attribute is the same with other attributes, then it is recommended to look at the next highest MSA inside the attributes that are tied and repeat until the tie is broken [16].

**Proposition 5** *If attributes are not dependent on each other then they are also not significant for each other*.

**Proof:** Eq (11) results 0 if attributes are not dependent on each other that is,

$$\begin{array}{c} k=\frac{\Sigma_{x\in U/Q}\left|\underline{P}\left(X\right)\right|}{\left|U\right|}=\frac{0}{\left|U\right|}=0. \end{array}$$ (13)

Now, Eq (12) of significance of attributes gives,

$$\begin{array}{c} \sigma_{Q}\left(P\right)=\gamma_{A^{{}^{\prime }}}\left(Q\right)-\gamma_{A^{{}^{\prime \prime }}}\left(Q\right)=\frac{\Sigma_{x\in U/A^{{}^{\prime }}}\left|\underline{P}\left(X\right)\right|}{\left|U\right|}-\frac{\Sigma_{x\in U/A^{{}^{\prime \prime }}}\left|\underline{P}\left(X\right)\right|}{\left|U\right|}=\frac{0}{\left|U\right|}-\frac{0}{\left|U\right|}=0, \end{array}$$ (14)

where A is set of all attributes and *A*′ = *A* − {*Q*}, *A*′′ = *A*′ − {*P*}. Hence proved that if attributes are not dependent on each other then they are also not significant for each other.

**Proposition 6** *Computational complexity of MIA is lower than that of MDA and MSA*.

**Proof:** Suppose that in an information system, there are *n* objects and *m* attributes. It requires *nm* computation for determining elementary indiscernibility relations for all attributes. MIA utilizes these elementary sets for selecting the best clustering attribute. Hence, the computational complexity for MIA technique is of the polynomial *O*(*nm*). For MDA, after computing elementary indiscernibility relations of all attributes it needs *n*(*n* − 1) times to determine the dependency degree of attributes. Thus, the computational complexity for MDA technique is of the polynomial *O*(*n*(*n* − 1) + *nm*). Also, for MSA, after computing elementary indiscernibility relations of all attributes it needs *n*(*n* − 1) + *n*(*n* − 2) times to determine the significance of attributes. Thus, the computational complexity for MIA technique is of the polynomial *O*(*n*(*n* − 1) + *n*(*n* − 2) + *nm*).

### 4.3 Evaluation metrics

The evaluation metrics purity and entropy are already defined in Section 3 whereas remaining measurse are presented in subsequent paras.

#### 4.3.1 Accuracy

The ratio between the number of correctly clustered objects over the total number of objects is accuracy [26]. After finding the true positive(TP), true negative(TN), false negative(FN) and false positive(FP), the accuracy is calculated as,

$$\begin{array}{c} Accuracy=\frac{TP+TN}{TP+FP+TN+FN} \end{array}$$ (15)

#### 4.3.2 Minimum Number of Iterations

As the number of iterations shows the computational complexity of desired technique so, lesser number of iteration to perform clustering task indicates better technique. Minimum steps required to find the value sets, indiscernibility relations, dependency, significance of each attribute and maximum values are counted as the iterations. For any technique, this evaluation shows the easiness with efficiency.

#### 4.3.3 Response Time

The response time of CPU to perform clustering task is examined by counting the time in milliseconds. Like wise the minimum number of iterations, response time also predicts the computational complexity of that technique. Hence, lesser time indicates a better technique. The rapidness with efficiency of any technique can be seen by this evaluation.

#### 4.3.4 Rough Accuracy

Mean roughness is used to measure the rough accuracy of selecting clustering attribute. The higher the mean roughness is the higher the accuracy of the selecting clustering attribute. The mean roughness of attribute *a*_*x*_*ϵA*, with respect to attribute *a*_*y*_*ϵA*, where *x* ≠ *y*, denoted *Rough*_*a*_*y*__(*a*_*x*_) is evaluated as follows,

$$\begin{array}{c} Rough_{a_{y}}\left(a_{x}\right)=\frac{\sum_{k=1}^{\left|V(a_{x})\right|}R_{a_{y}}\left(X|a_{x}=b_{k}\right)}{\left|V(a_{x})\right|} \end{array}$$ (16)

## 5 Experimentation and Comparison

An empirical study is performed on various small cases and six UCI data sets for categorical clustering using MDA, MSA and MIA techniques. The UCI data sets includes Lenses (24 instances, 4 attributes), Hayes-Roth (132 instances, 5 attributes), Molecular biology-Splice (3190 instances, 62 attributes), Balloons (16 instances, 5 attributes), Train (10 instances, 32 attributes) and Soya been (47 instances, 35 attributes). The comparison process of finding best clustering attribute using these techniques were organized in the light of research questions in Section 1 and in terms of different cluster evaluation parameters like purity and entropy. Moreover, the effect of the nature of data sets over the performance of these clustering technique is also analyzed. The reason behind taking various data sets is to investigate the generalizabilty ability and performance of these techniques on different data sets. Now in subsequent subsections, the research questions are separately analyzed and discussed in detail. To illustrate procedure of selecting best clustering attribute by MDA, MSA and MIA techniques, the dependency, significance degree and indiscernibility relation cardinality of an information system are computed step wise only in first example of Section 5.1.1.

### 5.1 Zero Dependency and Significance Degree

The cases where attributes are not dependent on each other, then automatically the resultant dependency degree of each attribute is zero. Similarly, if each attribute has no significance on remaining attributes then significance degree produced by each attribute is also zero. In this situation, the MDA and MSA techniques faces difficulty to select or failed to select the best clustering attribute as one cannot select maximum among all zeros. On the other hand, the MIA technique successfully select best clustering attribute even if attributes are not dependent or not significance for each other. Two cases are discussed here. Case 1 is Dengue Diagnosis information system taken from [27] while, Case 2 is Lenses data set taken from the UCI repository. The results of these examples also helps in validating Proposition 5.

#### 5.1.1 Dengue Diagnosis

Patients with possible dengue symptoms are presented in Table 1, which is taken from [27]. In this data set, twenty patients were considered having three symptoms or categorical attributes: Symptom A(SYMP A), Symptom B(SYMP B) and Symptom C(SYMP C).

**Table 1:** A Dengue Diagnosis Information System.

Patient	SYMP A	SYMP B	SYMP C	Decision
a	No	No	Normal	1
b	No	No	High	1
c	No	No	Very High	0
d	No	Yes	High	0
e	No	Yes	Very High	0
f	Yes	Yes	High	0
g	Yes	Yes	Very High	0
h	No	No	High	1
i	Yes	No	Very High	0
j	Yes	No	High	1
k	Yes	No	Very High	1
l	No	Yes	Normal	1
m	No	Yes	High	0
n	No	Yes	Normal	1
o	Yes	No	Normal	1
P	Yes	No	Normal	1
q	Yes	No	High	1
r	Yes	Yes	Very High	0
s	Yes	No	Normal	1
t	No	Yes	Normal	1

Firstly, the equivalence classes induced by indiscernibility relation of singleton attributes are obtained. In the next step, procedures to find dependency, significance and indiscernibility relation cardinality of each attribute are presented. The dependency of attributes of each data set is evaluated using Eq (11), whereas the significance of each attribute with respect to other attributes can be computed via Eq (12). Similarly, the cardinality of indiscernibility relations can be computed using Eq (5). From Table 1, based on each attribute, there are three partitions of U induced by indiscernibility relation on each attribute.

U/SYMP A = {(*a*, *b*, *c*, *d*, *e*, *h*, *l*, *m*, *n*, *t*), (*f*, *g*, *i*, *j*, *k*, *o*, *p*, *q*, *r*, *s*)}

U/SYMP B = {(*a*, *b*, *c*, *h*, *i*, *j*, *k*, *p*, *q*, *s*), (*d*, *e*, *f*, *g*, *l*, *m*, *n*, *o*, *r*, *t*)}.

U/SYMP C = {(*b*, *d*, *f*, *h*, *j*, *m*, *q*), (*c*, *e*, *g*, *i*, *k*, *r*), (*a*, *l*, *n*, *o*, *p*, *s*, *t*)}.

Based on Eq (11), the degree of dependency of attribute SYMP A on SYMP B, denoted as SYMP B ⇒ SYMP A, can be calculated as follows,

*SYMPB* ⇒_*k*_ *SYMPA*,

$$\begin{array}{c} k=\frac{\Sigma_{x\in U/SYMPA}\left|\underline{SYMPB}\left(X\right)\right|}{\left|U\right|}=\frac{\left|\{\}\right|}{\left|\{a,b,...,t\}\right|}=0. \end{array}$$

Using the same way we obtain,

*SYMPC* ⇒_*k*_ *SYMPA*,

$$\begin{array}{c} k=\frac{\Sigma_{x\in U/SYMPA}\left|\underline{SYMPC}\left(X\right)\right|}{\left|U\right|}=\frac{\left|\{\}\right|}{\left|\{a,b,...,t\}\right|}=0. \end{array}$$

Table 2 summarized the degree of dependency of all attributes of dengue diagnosis information system. It shows that the MDA technique have not been able to select a clustering attribute because maximum dependency degree of each attribute is 0. Thus, the MDA technique would lead a problem, because all the values are same that is 0.

**Table 2:** Dependency Degree of Attributes for Dengue Diagnosis Information System.

Attribute	Dependence		MDA
SYMP A	SYMP B	SYMP C	0
	0	0	
SYMP B	SYMP A	SYMP C	0
	0	0	
SYMP C	SYMP A	SYMP B	0
	0	0	

We can get the significance of subsets of U based on each attribute with respect to other attributes via Eq (12).

**i-**The significance of attribute SYMP A with respect to attribute SYMP B, denoted as *σ*_*SYMPB*_(*SYMPA*), can be calculated as follows.

Let *C*′ represents all attributes except attribute SYMP B that is *C*′ = {*SYMPA*, *SYMPC*}.

And *C*′′ = *C*′ − {*SYMPA*} = {*SYMPC*}.

*U*/*C*′ = {(*a*, *l*, *n*, *t*), (*b*, *d*, *h*, *m*), (*c*, *e*), (*f*, *j*, *q*), (*g*, *i*, *r*, *k*), (*o*, *p*, *s*)}.

*U*/*C*′′ = {(*b*, *d*, *f*, *h*, *j*, *m*, *q*), (*c*, *e*, *g*, *i*, *k*, *r*), (*a*, *l*, *n*, *o*, *p*, *s*, *t*)}

$\sigma_{SYMPB}\left(SYMPA\right)=\gamma_{C^{\prime }}\left(SYMPB\right)-\gamma_{C^{\prime \prime }}\left(SYMPB\right)=\frac{0}{20}-\frac{0}{20}=0$.

**ii-**The significance of attribute SYMP A with respect to attribute SYMP C, denoted as *σ*_*SYMPC*_(*SYMPA*), can be calculated as follows.

Let *C*′ represents all attributes except attribute SYMP C that is *C*′ = {*SYMPA*, *SYMPB*}.

And *C*′′ = *C*′ − {*SYMPA*} = {*SYMPB*}.

*U*/*C*′ = {(*a*, *b*, *c*, *h*), (*d*, *e*, *l*, *m*, *n*, *t*), (*f*, *g*, *o*, *r*), (*i*, *j*, *k*, *p*, *q*, *s*)}.

*U*/*C*′′ = {(*a*, *b*, *c*, *h*, *i*, *j*, *k*, *p*, *q*, *s*), (*d*, *e*, *f*, *g*, *l*, *m*, *n*, *o*, *r*, *t*)}

$\sigma_{SYMPC}\left(SYMPA\right)=\gamma_{C^{\prime }}\left(SYMPC\right)-\gamma_{C^{\prime \prime }}\left(SYMPC\right)=\frac{0}{20}-\frac{0}{20}=0$.

The resultant significance degrees for all attributes are presented in Table 3. Based on this table, the MSA technique have not been able to select the best clustering attribute, because the maximum significance degree for each attribute is 0. Moreover, one cannot choose maximum among all zeros, thus the MSA technique would lead a problem for this case.

**Table 3:** Significance Degree of Attributes for Dengue Diagnosis Information System.

Attributes	Significance		MSA
SYMP A	SYMP B	SYMP C	0
	0	0	
SYMP B	SYMP A	SYMP C	0
	0	0	
SYMP C	SYMP A	SYMP B	0
	0	0	

On the other hand, the MIA technique doesn’t require dependency or significance among attributes hence, it successfully select best clustering attribute for this data set. Using Eq (5),

$$\begin{array}{c} card(IND(SYMPA))=|IND(SYMPA)|=2 \end{array}$$ (17)

The indiscernibility relations cardinality for each attribute is presented in Table 4. According to this table, the attribute SYMP C has maximum cardinality of its indiscernibility relation, hence it is most indiscernible attribute and by MIA technique it is selected as best clustering attribute.

**Table 4:** Indiscernibility Relations Cardinality for Table 1.

Attribute(s)	Indiscernibility Relations Cardinality	MIA
SYMP A	2	-
SYMP B	2	-
SYMP C	3	3

#### 5.1.2 Lenses

UCI Lenses data set comprises of 24 instances and 4 conditional attributes. The degree of dependencies of all attributes of Lenses data set are summarized in Table 5. While, the results of the significance of all attributes is presented in Table 6. It can seen in both tables that for each attribute, the maximum dependency and significance value is 0. Hence, here both MDA and MSA techniques fails to select best clustering attribute based on maximum dependency and significance degree. Whereas, the MIA technique irrespective of attribute dependency or significance, selects best clustering attribute for on basis of maximum indiscernibility relations cardinality. For each attribute, the indiscernibility relations cardinality is presented in Table 7. which shows, the attribute 1 has higher indiscernibility relation cardinality that is 3. Hence, it is most indiscernible attribute and by MIA technique it is selected as best clustering attribute.

**Table 5:** Dependency Degree of Attributes for Lenses Data Set.

Attribute	Dependence			MDA
A	B	C	D	0
	0	0	0	
B	A	C	D	0
	0	0	0	
C	A	B	D	0
	0	0	0	
D	A	B	C	0
	0	0	0	

**Table 6:** Significance Degree of Attributes for Lenses Data Set.

Attributes	Significance			MSA
A	B	C	D	0
	0	0	0	
B	A	C	D	0
	0	0	0	
C	A	B	D	0
	0	0	0	
D	A	B	C	0
	0	0	0	

**Table 7:** Indiscernibility Relations Cardinality for Lenses Data Set.

Attribute(s)	Indiscernibility Relations Cardinality	MIA
Attribute 1	3	3
Attribute 2	2	-
Attribute 3	2	-
Attribute 4	2	-

### 5.2 Same Dependencies and Significance Degrees

There are cases where two or more attributes of any data set are equal dependent or significant for each other. As a result for these data sets, the MDA and MSA techniques gives the same maximum dependence and significance degrees respectively. In this situation, both techniques finds difficulty in selecting or unable to select an attribute as their best clustering attribute. The reason is simple that one cannot select maximum among same values. On the other hand, the MIA technique successfully select best clustering attribute even if attributes are equally dependent or equally significant. To explain this limitation of MSA and MDA, three example case studies Suraj’s LEMS [28], Pawlak’s Car performance [29]and Grzymala’s inconsistent [30] data sets are discussed here.

#### 5.2.1 Suraj’s LEMS Data Set

Lets consider a very simple information system shown in Table 8 taken from [28]. The set of objects U consists of seven objects with two conditional attributes: Age and LEMS (Lower Extremity Motor Score) and one decision attribute (Walk).

**Table 8:** Suraj’s LEMS Data Set.

	AGE	LEMS	WALK
*x*_1_	16-30	50	Yes
*x*_2_	16-30	0	No
*x*_3_	31-45	1-25	No
*x*_4_	31-45	1-25	Yes
*x*_5_	46-60	26-49	No
*x*_6_	16-30	26-49	Yes
*x*_7_	46-60	26-49	No

Table 9 summarizes the degree of dependency of all attributes of LEMS data set for MDA technique. Looking into this table, the maximum dependency degrees of AGE and LEMS attributes are equal that is 0.5714. Selecting maximum among same values is impossible, therefore the MDA technique would lead a problem in selecting best clustering attribute. Similarly, for MSA technique after computing the significance degrees of all attributes the results are summarized in Table 10. The MSA technique also faces difficulty in selecting best clustering attribute as maximum significance degrees for AGE and LEMS attributes are equal that is 0.5714. Thus, likewise MDA the MSA technique also not been able to select best clustering attribute for this data set. Whereas, by considering MIA technique the best clustering attribute is successfully selected. Table 11 presents the indiscernibility relation cardinality for each attribute of Suraj’s LEMS data set. The MIA technique selects LEMS attribute as best clustering attribute because the LEMS attribute has higher indiscernibility relation cardinality that is 4.

**Table 9:** The degree of dependency of attributes from Suraj’s LEMS Data Set.

Attribute	Dependence	MDA
AGE	LEMS	0.5714
	0.5714	
LEMS	AGE	0.5714
	0.5714	

**Table 10:** The degree of significance of all attributes from Suraj’s LEMS Data Set.

Attribute	Significance	MSA
AGE	LEMS	0.5714
	0.5714	
LEMS	AGE	0.5714
	0.5714	

**Table 11:** Indiscernibility Relations Cardinality from Suraj’s LEMS Data Set.

Attribute(s)	Indiscernibility Relations Cardinality	MIA
AGE	3	-
LEMS	4	4

#### 5.2.2 Grzymala’s Information System

Considering Grzymala’s information system from [30] as shown in Table 12. This is a patient data set having a decision attribute and four conditional attributes i.e. A, B, C, D expressing certain symptoms of a disease. Taking into account the MDA technique, which required the degree of dependency of all attributes of Grzymala’s data set. The computed degree of dependencies are summarized in Table 13. This table shows that the attributes B, C and D has first(0.16), second(0) and last(0) maximum dependency degrees same. Thus, the MDA technique unable to select best clustering attribute among attributes B, C and D as all possible maximum dependencies values of these attributes are equal. However, for this data sets the MIA technique can select the best clustering attribute on basis of maximum indiscernibility relation cardinality. The indiscernibility relation cardinality for each attribute of Grzymala’s information system is illustrated in Table 14. Attribute A has comparatively higher indiscernibility relation cardinality that is 3, hence it is selected as best clustering attribute using MIA technique.

**Table 12:** Grzymala’s Information System.

Case	A	B	C	D	Decision
1	high	yes	no	yes	1
2	v.high	yes	yes	no	1
3	high	no	no	no	0
4	high	yes	yes	yes	1
5	normal	yes	no	no	0
6	normal	no	yes	yes	0

**Table 13:** The degree of dependency of attributes from Grzymala’s Information System.

Attribute	Dependence			MDA
A	B	C	D	-
	0	0	0	
B	A	C	D	0.16
	0.16	0	0	0
				0
C	A	B	D	0.16
	0.16	0	0	0
				0
D	A	B	C	0.16
	0.16	0	0	0
				0

**Table 14:** Indiscernibility Relations Cardinality from Grzymala’s Information System.

Attribute(s)	Indiscernibility Relations Cardinality	MIA
A	3	3
B	2	-
C	2	-
D	2	-

#### 5.2.3 Pawlak’s Car performance Data Set

Pawlak’s Car performance data set in Table 15 is taken from [29]. There are six cars (m = 6) with three(n = 3) conditional attributes i.e. *a* = Terrain familiarity, *b* = Gasoline level, *c* = Distance. Considering the MSA technique, the significance degrees of all attributes are summarized in Table 16. This table shows that attribute *b* and attribute *c* has first(1) and second(0.67) maximum same. Due to same maximum significance degrees so, the MSA technique faces a problem in selecting best among both attributes *b* and *c*. Whereas, the MIA technique successfully selects best clustering attributes on basis of maximum indiscernibility relation cardinality. Table 17 presents the indiscernibility relation cardinality for each attribute of Pawlak’s Car performance data set. According to which, the attribute *b* and *c* has higher but equal indiscernibility relation cardinality that is 3. Hence, according to MIA technique the possible combinations of *b* and *c* attributes will be taken. The indiscernibility relation cardinality for only possible combination(*b*+*c*) is 4 which is maximum. Hence, this resultant combination of attributes that is b+c is selected as best clustering option using MIA technique.

**Table 15:** Pawlak’s Car performance Data Set.

U	a	b	c	d
1	poor	low	short	<30
2	poor	low	short	<30
3	good	low	medium	<30
4	good	medium	short	30…50
5	poor	low	short	<30
6	poor	high	long	>50

**Table 16:** The degree of significance of all attributes from Pawlak’s Car performance Data Set.

Attribute	Significance		MSA
a	b	c	-
	0.67	0.67	
b	a	c	1
	0.67	1	0.67
c	a	b	1
	0.67	1	0.67

**Table 17:** Indiscernibility Relations Cardinality from Pawlak’s Car performance Data Set.

Attribute(s)	Indiscernibility Relations Cardinality	MIA
a	2	-
b	3	-
c	3	-
b+c	4	4

### 5.3 Selecting Different Attributes as Best

If MIA, MSA and MDA techniques in some cases select different attribute as their best clustering attribute, then evaluations measures results also differently. From Proposition 6, it can be concluded that MIA technique is taking lesser iterations and time to select the its best clustering attribute as compare to MDA and MSA techniques. The results also proves that the MIA technique outperforms other techniques for evaluation measures like accuracy, purity, rough accuracy and entropy. Moreover, as compare to MSA technique the MDA technique selects their best clustering attribute in lesser iterations and response time whereas, the MSA technique shows better results for remaining evaluation measures than MDA technique. Five UCI data sets are utilized here for experimentation. That includes Hayes-Roth, Splice, Balloons, Train and Soya been.

**Fig 2:**
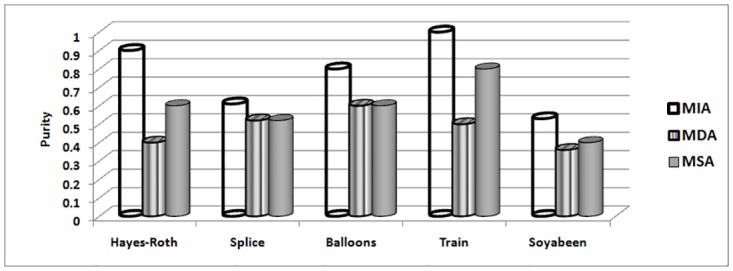
Purity for UCI data sets.

**Fig 3:**
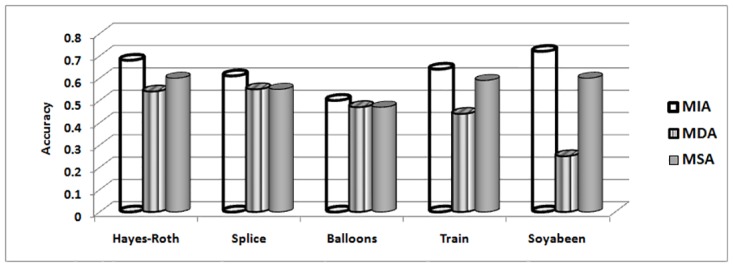
Accuracy for UCI data sets.

**Fig 4:**
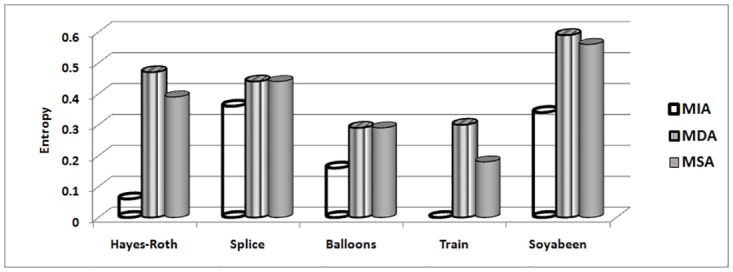
Entropy for UCI data sets.

**Fig 5:**
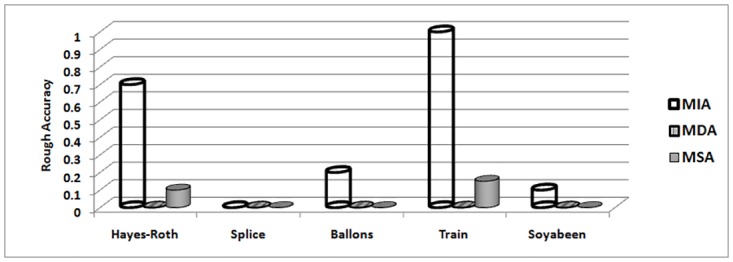
Rough accuracy for UCI data sets.

The MIA, MDA and MSA techniques selects their best attribute on basis of maximum indiscernibility relation cardinality, dependency and significance degrees respectively. In terms of respond time and minimum iterations, Tables 18 and 19 illustrates the results. It can be seen that MIA performs better due to less iteration required and better response time as compare to other techniques for all data sets. The number of iterations includes steps for finding maximum dependency among attributes for MDA, significance of attributes for MSA and cardinality of indiscernibility relations of attributes for MIA technique. Considering remaining evaluations measures like purity Fig 2, accuracy Fig 3, entropy Fig 4 and rough accuracy Fig 5, the MIA technique also proves to be comparatively better and efficient for these data sets. It can also be seen from Tables 18 and 19 that the MDA utilizes less iterations and time than MSA in selecting their best clustering attribute. However, for remaining all evaluation measures as presented in Figs 2–5 the MSA outperformed MDA.

**Table 18:** Response time of techniques.

Data Set	Response time(millisec)		
	MDA	MSA	MIA
**Hayes-Roth**	6	8	**0**
**Splice**	510	1679200	**31**
**Balloons**	0	0	**0**
**Train**	5	26	**1**
**Soya been**	41	219	**0**

**Table 19:** Minimum iteration consumed by techniques.

Data Set	Response time(millisec)		
	MDA	MSA	MIA
**Hayes-Roth**	76	527	**8**
**Splice**	86531	11481559	**63**
**Balloons**	80	147	**11**
**Train**	5196	10272	**33**
**Soya been**	31877	57517	**36**

## 6 Discussion

The research questions that are designed in start of this article are answered and discussed in this section. The answers are based on the results of experiments performed in Section 5. The first two questions deals with the limitations of MDA and MSA techniques in selecting best clustering attribute whereas the last research questions explores the pros and cons of both techniques. Finally, the performance of alternative novel technique MIA is discussed for each research question in light of limitations, pros and cons of MDA and MSA techniques.

1. What if attributes have zero dependency and significance degree?
  The results presented in Section 5.1 shows that the MDA and MSA techniques are unable to select their best clustering attribute whenever the maximum degrees of dependency and significance are all zeros. In such cases, the attributes are not dependent on each other due to which the attributes have zero dependency degrees. Whereas, the zero significance for all attributes means that in these cases the presence or absence of an attribute has no effect on other attributes. Also, interestingly it is explored that if attributes are not dependent on each other then they are not significance for each other. This situation lead to a useful limitation of MDA and MSA technique, that both techniques cannot select best clustering attribute if attributes are not dependent or attributes have no significance on each other. Whereas, the proposed MIA technique only require the indiscernibility relations for selecting clustering attribute and it doesn’t depend on dependency or significance among attributes. So, MIA technique successfully selects best clustering attribute even if the attributes are not dependent on each other.
2. What if attributes have same degree of dependencies or significance?
  The outcomes of cases discussed in Section 5.2 concludes that if all possible maximum dependency and significance degrees of two or more attributes are coming same, then the MDA and MSA fails to select their best clustering attribute. Actually, those attributes are giving first, second and up to last maximum degrees same and selection of maximum value among similar values is impossible. This case of resulting similar dependency or significance degrees of attributes leads to another useful limitation of MDA and MSA techniques. Meanwhile, the proposed MIA technique works only on domain knowledge of any data set in form of indiscernibility relations for selecting clustering attribute, hence it doesn’t requires any dependency or significance among attributes. Therefore, MIA technique successfully selects best clustering attribute even if the attributes have similar dependence or significance degrees.
3. What if the techniques select different attributes as their best clustering attribute?
  Section 5.3 illustrates that the MIA technique outperformed MDA and MSA techniques for all evaluation measures in the process of selecting best clustering attribute. Moreover, the results also concludes that both MDA and MSA techniques have certain pros and cons over each other. Like for example, the MDA technique is good in terms of response time and minimum iterations required for finding its best clustering attribute. Whereas, the MSA technique despite of having more computational steps but its performance is better in terms of accuracy, purity, rough accuracy and entropy. Meanwhile, the proposed MIA technique not only perform better in terms of number of iterations and response time but also it proves to be more efficient than MDA and MSA in terms of purity, entropy rough accuracy and accuracy.

## 7 Conclusion

An alternative rough categorical clustering technique MIA is proposed in light of some useful limitations, pros and cons of existing techniques like MDA and MSA. The effect of special data sets nature over the performance of MDA and MSA clustering techniques is analyzed to explore the difficulties and issues faced by both techniques in selecting their best clustering attributes. Moreover, this work illustrates how MIA is resolving those issues. MIA technique utilizes rough indiscernibility relations of each attribute in selecting its best clustering attribute. The MIA technique is proven to be better, efficient, simple and more general as compare to MDA and MSA clustering techniques in terms of number of iterations, response time, purity, entropy, rough accuracy and accuracy. Ten different data sets from UCI repository and previously used research cases are utilized for experiments. Moreover, this study provides the users an alternative approach for selecting a proper and effective rough clustering technique to select best clustering attribute. The performance of the proposed MIA technique shows that it can be extended for other real and big categorical data sets.
